# End-of-Life Care Education as Blended Learning Approach for General Practitioners: a Scoping Review

**DOI:** 10.1007/s13187-023-02358-w

**Published:** 2023-08-30

**Authors:** Shrikant Atreya, Naveen Salins

**Affiliations:** 1 Department of Palliative Care and Psycho-Oncology, Tata Medical Center, Kolkata, West Bengal 700160 India; 2 Department of Palliative Medicine and Supportive Care, Kasturba Medical College, Manipal, Manipal Academy of Higher Education, Manipal, Karnataka 576104 India

**Keywords:** End-of-life care, Education, Training, General practitioners

## Abstract

General practitioners (GPs) are critical in providing primary palliative care in the community. Apprehensions about managing a dying person at home, difficulties in goals of care discussion, limited resources and lack of palliative care education often hinder end-of-life care provision in the community. This review focused on the end-of-life care training programs accessed by GPs and sought to understand if the training programs’ content and mode of delivery aligned with their preferred needs. MEDLINE, EMBASE, CINAHL, and PsycINFO were searched to identify articles published in English between 01 January 1990 and 30 September 2022. Additionally, searches were conducted using SCOPUS, the Web of Science, and the Cochrane database using free texts. The reviewers screened the titles, abstracts, and full text to identify eligible studies and extracted textual data to analyse and generate themes. Out of 5532 citations initially accessed, 17 studies were included in the review. Six themes were generated: knowledge translation, skill development, a change in attitude, self-efficacy, satisfaction, and patient outcomes. The GPs’ end-of-life care knowledge, skills, attitude, self-efficacy, and patient outcomes were better when their training had a combination of small-group interactive workshops, trigger case-based reflective learning, mentor-facilitated experiential learning, web-based modules, and peer learning. The synthesis of review findings supports blended learning as a training approach for general the practitioners’ end-of-life care education as it facilitates learning and patient outcomes.

**Supplementary Information:**

The online version contains supplementary material available at 10.1007/s13187-023-02358-w.

## Introduction

Worldwide, over 56.8 million people need palliative care annually, and 76% reside in low- and middle-income countries (LMIC) [1]. Palliative care needs, estimated using the prevalence of serious health-related suffering, are projected to increase by 87% in 2060 [2]. However, it is challenging to know the number of people accessing palliative care due to a lack of national databases in most parts of the world [3]. Home-based palliative care reduces hospital utilisation [4] and enhances the quality of life in patients with cancer [5] and heart failure [6] whilst improving patient and caregiver satisfaction [5, 6]. However, patients’ preference for home-based care often depends on availability [5]. General practitioners are critical to delivering primary palliative care in the community [7]. They are well-positioned to ensure patient coordination and continuity of care at the end of their lives [7]. Despite their role in end-of-life care, they lack confidence in managing pain and physical symptoms, addressing patients’ and caregivers’ psychological needs, conducting goals of care discussions, and facilitating advance care planning [8, 9]. These apprehensions may negatively influence end-of-life care provision in the community and mandate urgent evidence-based end-of-life care training [10, 11].

Two systematic reviews have been published in 2006 and 2020 that focused on end-of-life care education for primary care physicians [12, 13]. The reviews included a mixed population of primary care physicians at various career trajectories, with a narrow focus on general practitioners providing end-of-life care in the community [12, 13]. It highlighted gaps in end-of-life care training, like goals of care discussion and advance care planning [9]. However, past reviews have not addressed GPs’ preferences for end-of-life care training, patient outcomes, and satisfaction. GPs will seek a training programme only if it aligns with their perceived value and is relevant to clinical practice [4]. Furthermore, there is a need to explore the impact of end-of-life care training on patient-reported outcomes, as it influences physician performance and improves adherence to recommended clinical practice guidelines [14].

The current review focused on scoping end-of-life care training programs for GPs, their learning preferences, and perceived outcomes. Furthermore, it also focused on the training programs’ content and mode of delivery and whether it aligned with their preferred needs [10].

## Methods

### Review Question

What types of end-of-life care education programs are accessed by general practitioners, and how do they impact clinical practice outcomes?

### Review Design

The review aimed to systematically synthesise and report the range of end-of-life care education programs accessed by general practitioners and their impact on clinical practice outcomes [15, 16]. A scoping review is conducted where there is a heterogeneous body of literature in an understudied field. Exploring them informs the breadth of published literature on the phenomenon explored [15, 16]. It also identifies gaps in the research and enables the identification of types and sources of evidence that inform practice, policy, and future research [15]. The studies were reported using the PRISMA ScR checklist extension for scoping studies [17]. The review adopted and combined the steps described by Arksey and O’Malley [16] for scoping reviews and the Levac et al. enhancements [18]. The Levac et al. [18] enhancements included identifying the research question in conjunction with the purpose of the review and clearly defining the context, concept, and population studied. It helps identify relevant studies, including the justification for limiting the scope of the search. It also facilitates charting the data using descriptive analysis as an ongoing process and collating results [18]. Moreover, the PAGER (patterns, advances, gaps, evidence for practice and research recommendations) framework was used as a reflective tool for analysing and reporting scoping reviews [19]. It helped enhance the rigour of the scoping reviews by providing a framework for consistently presenting the findings [19].

### Search Strategy

Databases like MEDLINE, EMBASE, CINAHL, and PsycINFO were searched to identify articles published in English between 01 January 1990 and 30 December 2022 (Supplementary File 1). Additionally, searches were conducted using SCOPUS, the Web of Science, and the Cochrane database using free texts. The search was performed using a thesaurus and free-text terms specific to the database, and the terms were combined using Boolean operators [20]. The bibliography of included studies was searched to identify additional relevant studies.

### Inclusion and Exclusion Criteria

Studies were included in the review if they met the eligibility criteria outlined in Table 1.

**Table 1:** Eligibility criteria

Inclusion criteria	
Focus of evidence	The focus was educational intervention in primary palliative care
	Studies that described different types of training programs
	Studies describing feedback, monitoring, and evaluation of educational intervention of trainees’ pre- and post-training or post-application of training in clinical practice
Population	A general practitioner providing community-based care
Setting	Community-based primary palliative care
Types of papers	Empirical research studies published in English
Date	Papers published from 01 October 1990 onwards
Exclusion criteria	
Population	Healthcare professionals other than GPs, healthcare assistants, specialist training in palliative care or palliative care fellowship programs, family or other informal caregiver training, and training of volunteers
Focus of evidence	Studies only limited to educational needs or learning preferences in end-of-life care
Type of papers	Conference abstracts, editorial papers, letters to editors, grey literature, and newspaper articles

### Data Extraction and Analysis

Citations from database searches were exported to a reference manager, and duplicates were removed. The two reviewers (SA and NS) screened titles, abstracts, and full text to identify eligible studies. Reviewers discussed conflicts regarding the eligibility of studies for inclusion in the review with an independent review supervisor. A data extraction sheet was used to tabulate and summarise information. The data included author(s), year of publication, country of origin, study aim, study design and sample, population characteristics, educational intervention, content, mode of delivery, and key findings.

## Results

### Overview of the Studies

**Fig. 1:**
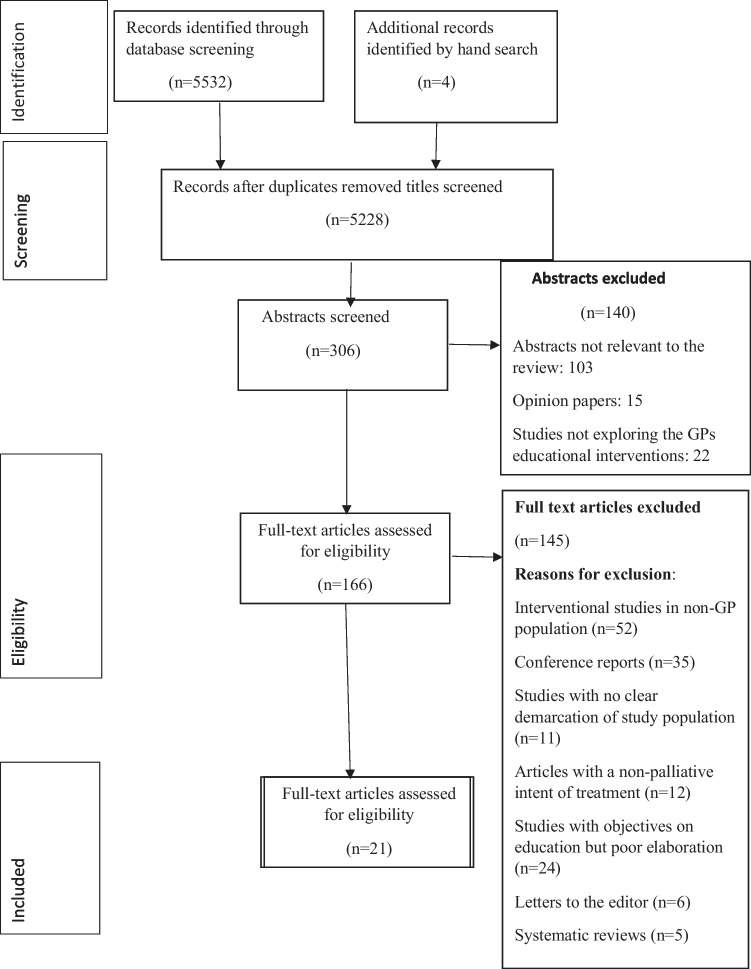
PRISMA SCR Flow Diagram for studies included in scoping review

Out of 5532 citations identified from database searches, 17 studies were included in the review (Fig. 1-PRISMA SCR flowdiagram). Additional four articles were identified after searching the bibliography of included studies [12, 13]. Of the 21 studies included, thirteen studies were before and after studies [21–33], six studies were randomised controlled trials [34–39], and two studies were quasi-experimental (Table 2, Supplementary File 2) [40, 41]. The before and after studies included objective assessments and qualitative data from in-depth interviews or focus group discussions (Table 3). Amongst the included studies for the review, four each were from Australia [21, 22, 28, 34], the Netherlands [30, 31, 38, 39], and Canada [23, 25, 27]. Two each from Spain [36, 37] and the UK [29, 32] and one each from Germany [41], Sweden [40], Denmark [35], New Zealand [26], Europe [33], and Finland [24].

**Table 2:** Overview of the studies included in the scoping review

Author (year) Country	Research objectives	Population (*n*)	Method	Interventions studied	Evaluation method	Outcome of intervention
Boakes et al. (2000) Australia [21]	To evaluate the effect of experiential training on palliative care skills of GPs	22 GPs	Before and after study	Weekly case conference: reflective learning under mentorship Experiential learning through engagement with patients on a weekly basis Rotation with hospice unit under a mentor Seminar sessions on symptom control, oncology, and psychosocial and ethical issues in palliative care	Process evaluation tool: overall satisfaction with the program satisfaction with various components of the program any aspects Survey to self-report (post-intervention) improvement in knowledge and skills Survey to self-report (post-6-month intervention) confidence in caring for patients	Knowledge: knowledge/skills questionnaire showed significant improvement in general palliative care management (*p* < 0.001) Confidence (post 6 months) Knowledge/skills questionnaire showed significant improvement in general palliative care management (*p* < 0.001)
Detering et al. (2014) Australia [22]	To assess the effect of ACP training program on confidence in undertaking advance care planning conversations with their patients The effect on performance on an advance care planning	148 GPs	Before and after study	Self-learning of materials in ACP DVD on case vignettes on ACP Patient e-simulation 2-h workshops and discussion	Self-reported confidence in ACP discussion, performance on the e-patient simulation, change in advance care planning knowledge and attitude, participants’ satisfaction, and participant’s willingness for future participation	There was significant improvement in confidence to have ACP discussion (*p* < 0.05) There was significant improvement in e-patient simulation scores (*p* < 0.001) There was significant improvement in ACP knowledge (*p* < 0.001) There was no significant change in the attitude before and after intervention. However, the participants post-intervention did feel that ACP discussion as less emotionally burdensome Participants were satisfied with workshop
Evans et al. (2021) Canada [23]	To enhance provider knowledge and confidence in palliative care delivery, ability to identify patients who might benefit from palliative care earlier in their disease trajectory, and to enhance the provision of palliative care and the use of palliative care tools	71 GP practice professionals	Before and after study	Interprofessional education: a 2-day workshop in interactive competency and team-based workshop Integrated approach to care: coordination of care in the community	A survey consisted of 20 questions on a Likert agreement scales was used to measure provider attitudes, confidence to provide palliative care, use of palliative care tools, and delivery of palliative care	Significant improvement in confidence in providing palliative care and use of palliative care tools (*p* < 0.05) Confidence improved in the following domains: (1) to provide palliative care, (2) use of surprise question, and (3) ACP discussion
Hinkka et al. (2002) Finland [24]	To evaluate the effect of the one- year educational project on attitudes, opinions and decisions of GPs	82 GPs	Before and after study	Educational materials Internet based interactive session 2-day seminar (face to face): case-based discussion and didactic lectures Role play Review of articles	Survey questionnaire Case vignettes VAS score for assessing attitude	Doctors in the intervention group were in favour of palliative care for cancer patients despite iatrogenic side effects (*p* = 0.025) and active treatment when family benefit was considered (*p* = 0.045) Most chose conservative approach decision against CPR in a terminal event (*p* = 0.004) including foregoing antibiotics, blood transfusions, hydration IG doctor’s attitude towards burnout changed with less of a problem in IG group although not statistically significant Satisfaction with profession (*p* = 0.037) and own health (*p* = 0.004) was stable in the IG as compared to CG
Kadlec et al. (2015) Canada [25]	GP satisfaction of the module—impact on their practice and EOL patients	608 GPs completed the EOL baseline survey, 381 completed the end-of-module survey, and 109 completed the 3–6-month follow-up survey	Before and after study	Practice-support program included an interactive session with interprofessional team of 3–4 face-to-face learning session (4 h each) interspersed with a 3–4-month-long experiential learning with GP champion or regional leader	Self-perceived satisfaction Perceived impact on practice EOL objective scale–practice change	At 3 months of module learning: Majority of the GPs were satisfied with the content of the sessions and mode of delivery, support received and applicability of training, and goals and measures to monitor the progress Physicians with greater than two-GP practice had highest satisfaction (*p* < 0.01) Women GPs rated the general impact of the EOL module on their practice, and their patients are higher than the men GPs perceived that the module helped bring changes in their practice and were more satisfied with their practice. This improved their patient care and also increased the knowledge of local resources. It improved their comfort in caring for EOL patients, improved collaborative work, and built trust and stronger relationship More experienced GPs gave lower objective ratings for the module At 3–6 months post-training: Although there was a significant rise in the GPs maintaining patient registry (from 7.9% at baseline to 65.9% at 3 months), but there was a drop by 13.5% at 6 months follow-up (*p* < 0.05) There was a significant rise in the number of GPs maintaining an action plan for the GPs from 28.3% at baseline to 68.9% at 3 months to 83.7% at 6 months (*p* < 0.001) There was a significant rise in the number of GPs accessing current palliative care guidelines from 61.7% at baseline to 88.8% at 3 months to 94.3% at 6 months (*p* < 0.001). There was, however, a decline in the collaborative work in the initial 3 months but did become more frequent during the follow-up period (*p* < .001) Although the frequency of home visits increased in the initial 3 months (*p* < 0.01), but there was no further rise at 6 months follow-up. physicians in two-GP practice conducted Confidence with EOL-related skills and knowledge There was a statistically significant (*p* < 0.001) improvement in the confidence of the physicians in identifying and initiating conversation on EOL with patient/family, support of patient/family in terminal event, goals of care discussion, collaborate with specialists/other teams, and support of family during bereavement phase
Landers et al. (2022) New Zealand [26]	To assess the confidence levels of GPs in EOLC before and after the delivery of a master class in palliative care Assessment of knowledge retention after 3 years of the course	22 GPs	Before and after study	4 workshops over 2 years—small-group interactive sessions (20/group) Case-based learning including sharing cases of experience Covered topics on opioids, symptom management, non-malignant palliative care, food, and fluids at the end-of-life and ethical dilemmas Interaction and case discussion with specialist palliative care team	Kirkpatrick four-level evaluation	There was significant improvement in the knowledge in symptom management (*p* < 0.05–0.01). However, topics that showed high degree of confidence but did not improve were S/C infusion, ethical dilemma, and ACP Post-3 years of training most applied the following in practice: BTP opioids, opioid management for pain, breathlessness and terminal event, managing constipation, and having ACP conversation
Marshall et al. (2008) Canada [27]	To understand the effect of the intervention on GP knowledge, skill and confidence in providing an interprofessional palliative care through a shared care model	12GPs	Before and after study	Style Interprofessional collaboration—15 practice-based discussions Shared care (between GP practice and specialist palliative care team) Weekly multidisciplinary meetings Problem-based learning approach Chart reviews Discussions on integrating practice with evidence-based approach Topics covered: Pain and symptom management Emergencies Prognostication Communication Team work	Patient outcomes: Preferred place of death Number of interprofessional collaborations GP perception of the project Survey, interviews, and FGDs	40% increase in the collaborative care 59% patients/families were cared through collaborative care 59% patients wish to die at home were fulfilled as compared to 28% before the study GPs perception: Contact with palliative care team as vital Practice-based education is valuable to maintain the role as a primary care provider is important Provision of comprehensive multidisciplinary care to patient as essential Nurses perception of coordinating with GPs Trust, respect, and confidence in care providers Improved communication Better anticipation of needs Sustainability of care Confidence in decision making Collaborative care as less stressful Adjustment in care provision
Reymond et al. (2005) Australia [28]	To improve the palliative care capacity of primary health care providers in rural communities	20 GPs and 98 other primary health care workers in the community	Before and after intervention study	3-h workshop Didactic lecture Small group case discussion Topics: pain management, pharmaceutical use, management of dyspnoea, delirium and constipation, care planning, dealing with families and grief and loss issues Psychosocial aspects	Cost effectiveness Educational outcomes—achievement of learning objectives and confidence in palliative care management Clinical outcomes—knowledge, skills, and management confidence (pre-workshop and 3 months post-workshop)	There was significantly perceived benefits that were reported for all specific knowledge items In items such as pain, dyspnoea, delirium, and constipation (mean score improvement of 4.1 (CI 3.5–4.6), 4.0 (CI 3.5–4.5), and 4.2 (CI 3.7–4.7), respectively) Improvement in skill at tailoring pharmacological management to patient needs (mean score improvement of 4.0 (CI 3.7–4.3) There was a statistically significant improvement in confidence in communication (3.3 (CI 2.9–3.8)) (*p* < 0.01) Evaluation at 3 moonths of workshop There was a statistically significant improvement in confidence in providing care in following domains: Nociceptive tissue pain (3.1, *p* < 0.02) Neuropathic pain (2.9, *p* < 0.01) Dyspnoea (2.9, *p* < 0.01) Constipation (3.3, *p* < 0.03) Delirium ( 2.8, *p* < 0.04)
Shipman et al. (2003) UK [29]	To evaluate the impact of the Macmillan GP facilitator program on knowledge, attitude, and confidence in symptom management, communication, and out-of-hours practice	449 GPs in quantitative study 63 GPs in pre-test qualitative interview 23 GPs in post-test qualitative interviews	Before and after study In-depth interview	Over 2 years Mentorship in the area of practice Educational visits by GP facilitators	Quantitative data was assessed on a 5-point Likert scale and binary data	Positive association between intervention and attitude on collaboration (*t* = 2.58; *p* = 0.01) in IG versus CG There was a statistically significant improvement in awareness about palliative day care facility, domiciliary care (younger GPs), and referral to specialist palliative care service (*p* = 0.04) An increase in confidence in controlling symptoms in patients with non-malignant disease was negatively associated with number of partners No significant differences were found for discussing diagnosis and prognosis with patients suffering from non-malignant disease Some associations were found between an increase in satisfaction with medical cover out-of-hours for palliative care patients and the facilitator intervention (*t* = 1.82, *p* = 0.07)
Thoonsen et al. (2016) Netherlands [30]	Views of both the GPs and the consultants who advised the GPs in order to fine-tune the proactive palliative care plan, 2 years after the GPs had been trained	13 GPs	Before and after study Focus group methods and individual telephone interviews	Intervention group: A 5-h group training in the early identification of palliative care patients Included individual coaching session over phone with a physician specialized in palliative care Two additional peer group sessions with the GPs in the intervention group a few months after the start of the intervention, with a focus on patient–GP communication regarding the initiation of a palliative care trajectory Control group: Usual care	Themes generated from qualitative interview	12 GPs felt a positive change in their attitude towards palliative care and were proactive in their care Majority of the GPs incorporated the indicators of identification of palliative care in their practice While a majority of GPs found communicating prognosis to patients difficult, some GPs did communicate to their patients about the anticipated future problems
Thoonsen et al. (2015) Netherlands [31]	Training would improve the care for palliative patients with cancer, COPD or CHF in the form of less contacts with the out-of-hours primary care cooperative, a decreased number of hospitalizations in the last 3 months of life, an increased number of contacts with their own GP in the last month of life, and an increased number of patients that would die at home	38 GPs–IG 39 GPs–CG	Cluster RCT	Intervention group: A 5-h group training in the early identification of those patients in their practice that can be considered as being palliative patients The use of means of the RADPAC indicator and in proactive care planning An individual coaching session by phone with a physician specialized in palliative care, per identified palliative patient for the GP Two additional peer group sessions with the GPs in the intervention group a few months after start of the intervention, with a focus on patient– GP communication regarding the initiation of a palliative care trajectory Control group: Usual care	Number of contacts with GP in the last month of life Number of hospitalisations Number of patients who would die at home	28 GPs identified 52 patients (0.91 per GP; 0–4) and in 33 cases the GP had an individual coaching session with the specialist in palliative care by phone (0.58 per GP) No differences between the intervention and control group in the number of contacts with the GP out-of-hours cooperative in that last three months, nor in the number of contacts a patient had with their own GP in the last month, hospitalisations in the last three months, dying at home, or dying in the hospital Identified patients had more contacts with their own GP in the last month of life (13.00 versus 7.48). Also the location of death differed: the identified patients died at home more often (67 versus 45%) and less often in the hospital (14 versus 32%) A smaller percentage of the identified patients had had at least one hospitalisation in the last three months of their life as compared to the other patients (42 versus 61). The mean number of hospital admissions of identified patients was also lower (0.60 versus 0.89) The number of contacts that identified patients had with their own GP in the last month before death (*p* = 0.0006). They were less often hospitalized in the last three months of life (*p* = 0.0437), and died less often in the hospital (*p* = 0.0449). Although they also died more often at home, this difference was not statistically significant (*p* = 0.0572)
Ward and Walsh (2009) UK [32]	To assess the effect of training on: Knowledge Confidence in palliative care delivery Satisfaction	8 GPs	Before and after interventional study	Independent study modules Experiential learning by rounding palliative care (2 OPDs and 2 hospice rounds) Face-to-face teaching sessions. a reflective ‘long case’ with a problem-solving approach (of patient they had cared for last 12 months)	Knowledge assessment using MCQ and short answer questions Self-rated confidence 5-point Likert scale	Median score on short answer question rose from 19/25 to 23/25 On a 5-point rating scale, the participants’ confidence at managing difficult symptoms in dying patients increased (median pre-course score 3, post-course 2) as did the confidence in dealing with difficult psychosocial problems when talking with dying patients (median pre-course 4, post-course 2) The participants’ use of a problem-solving approach (5 never, 1 always) during the course increased (median pre-course 3, post-course 2) By the end of the course, the participants’ perceived coping mechanisms to deal with the difficulties in caring for dying patients and their families improved Overall satisfaction with the intervention was high More GPs expressed confidence in handling communication in psychosocial aspects, better approach to difficult communication, discussing end-of-life care with patients, and perceived improvement in symptom control
Xhixha et al. (2013) Europe [33]	To evaluated the attitudes of family doctors on pain assessment, management, and opioid usage before and after seminars on opioid pain management	189 GPs	Before and after study	1-day seminars were scheduled for 8 h, including case-based discussion, interactive discussions, and debates	BQII standardised questionnaire	There was a 26% reduction in the barriers related to pain management (*p* < 0.001) such as managing the adverse effects of opioids A 38% increase in general palliative care (*p* < 0.01)
Abernethy et al. (2013) Australia [34]	Null hypotheses were that the addition of case conferences (study 1), GP education (study 2), and patient/caregiver education (study 3) would not influence pain, performance status, or health service utilization		Cluster (2X2X2) randomisation Cluster 1: case conference(in physical or telephonically) Cluster 2: educational material, traditional educational outreach Cluster 3: patient/caregiver education	Case conference (telephone/physical) Traditional educational outreach	Brief pain inventory MCGill QOL questionnaire Hospital utilisation Assessed at baseline, 2 weeks later, and monthly until death/withdrawal	Hospitalisation rates reduced significantly in cluster 1 (*p* = 0.0069) No significant reduction was observed in other clusters There was a marginal improvement in performance status (57.3 vs 51.7) (*p* = 0.0368), but no significant difference between groups in other 2 clusters The impact of patient/caregiver educational visiting for pain management approximated that of case conferences for people with lower performance status (1.58 vs 1.57) Patients in the interaction groups cluster 1 and 2 had significant improvement in the performance (*p* = 0.0216) Cluster 3 demonstrated significant reduction in symptom burden as compared to the other 2 clusters
Guldin et al. (2013) Denmark [35]	Effect of bereavement management program: Identification of complicated grief Management of complicated grief	167 GPs	Cluster-randomized controlled trial Intervention arm—educational material (pamphlets) Control group—routine care	Information pamphlets (CG symptoms, dual process model for coping, risk factors of CG, and how to assess CG and manage)	Tools for assessment: Beck’s Depression Inventory II (BDI-II)16 Inventory of Complicated Grief-Revised (ICG-R)	No statistically significant improvement was observed in the ICG-R in the intervention arm at 6 months but reversed at 13 months Also, there was greater improvement in the BDI_II score for mild/moderate depression at 6 and 12 months in IG as compared to CG, the severe depression group had marginal improvements Positive predictive value in IG—34.6% and negative predictive value—80% Counselling support provided was higher in IG (RR = 1.6) as compared to CG (RR = 0.8) (not statistically significant) Marginally lower diagnostic difficulty post-intervention in IG (8/18) versus CG (7/14) Referral to mental health profession was higher in IG (*p* < 0.01) Lower probability of psychotropic medications prescribing in IG compared to CG (*p* < 0.001)
Pelayo et al. (2011) Spain [36]	Effectiveness of online palliative care training on knowledge and attitude Perceived confidence in symptom management and communication GPs satisfaction post-training	164 GPs IG 82 CG 82	Randomised controlled trial IG—online platform of learning CG—traditional learning	Online training Comprising—educational material accessed over the period of training over 75 days Two tutors to facilitate the training	Assessment using 5-point Likert scale Knowledge and attitude toward PC Confidence in symptom management and communication	There was a significant improvement in knowledge (14–20%) and confidence in communication(35% IG versus 7% CG—*p* = 0.007) and not in symptom management (*p* = 0.151) in the IG versus CG In both the groups, a subgroup analysis of participants who had some training in PC in the past had marginal difference in the most useful aspects pointed out in terms of online training (50 participants (83.3%)) which were: practical, clear and systematic approach, with elaborated and updated materials; symptom management, death management, communication, opioid management; bibliography and websites; and tutoring and communication among participants
Pelayo-Alvarez et al. (2013) Spain [37]	To assess the impact of this training in symptom control on patients with advanced cancer The assessment of patient’s QOL, caregiver satisfaction, PCPs’ level of knowledge, and PCPs’ attitude toward PC and satisfaction post-intervention and at 18 months	66 GPs enrolled 117 patients 63 patients in the IG 54 patients in the CG	Randomised controlled trial IG-online platform of learning CG-traditional learning	Online training Comprising—educational material accessed over the period of training over 75 days Two tutors to facilitate the training	Patient outcomes: Spanish version of brief pain inventory(BPI) Palliative Outcome scale (POS) QOL was assessed using Rotterdam symptom checklist (RSCL) Caregiver satisfaction using the Spanish version of SERVQUAL	There was non-statistically significant reduction in pain score in IG on BPI, POS, and RSCL Caregiver satisfaction score in both the groups ranged between 3.2 and 4.2 and were statistically non-significant However, there was a significant reduction in the family anxiety in the IG Significant reduction in the global QOL scale on the RSCL in the IG versus CG There was significant improvement in PC knowledge in IG (positive difference of 5.2, CI 3.4–6.9) as compared to CG At 18 months, IG showed significant mean difference in PC knowledge over the control group (3.6 [95% CI, 2.0–5.2]; *p* = 0.0001) Confidence in patient symptom management and confidence in communication of diagnosis and disease prognosis showed no significant difference between groups at 18 months
Slort et al. (2014) Netherlands [38]	(1) Palliative care outcome measures, (2) satisfaction with the communication with their GP, and (3) ratings of their GP’s availability, and discussion of current and anticipated issues	126 GPs	Controlled trial 126 GPs where 62 GPs were assigned to the intervention group and 64 GPs to CG	Availability, current issues and anticipation (ACA) training programme for GPs comprised: Videotaped GP–patient (simulation) evaluation pre- (baseline) and post-training (at 6 months) Peer small group interaction under a specialist supervision Roleplay	Patient outcomes: The Palliative Care Outcome Scale (POS) The European organisation for research and treatment of cancer quality of life questionnaire core 15 palliative (EORTC QLQ-C15-PAL) The rest & peace scale (RPS) Patient satisfaction questionnaire–III (PSQ-III), The ACA scale measures the extent to which the GP was available for and discussed important issues with the patient	There was no significant reduction in the scales between groups noted The lower scores we found in both groups for one RPS and a few ACA items suggest that GPs might take more initiative to discuss the following end-of-life issues: unfinished business, prognosis and possible complications, the actual process of dying, including the preferred place of death, and end-of-life decisions
Tilburgs et al. (2020) Netherlands [39]	Effect of training on the initiation of ACP and the number of medical and nonmedical preferences discussed Cost-effectiveness analysis and studied the intervention’s effects on patient’s QoL and family carer’s sense of competence	38 GPs who further contacted patients 71 (patients/CG)–IG 63 (patients/CG)–CG	Single-blinded cluster-randomized controlled trial	Intervention group: Two 3-h interactive workshops Simulation with live patients on ACP discussions Educational material on ACP 2 monthly telephonic follow-up on GPs Control group: information booklet and usual care	proportion of PWD for whom ACP was initiated during the 6 months following the intervention Number of medical and nonmedical preferences discussed during all ACP conversations during the 6 months following the intervention Secondary outcomes were QoL (dementia, quality of life questionnaire, and Euro QoL 5D questionnaire), experienced level of SDM of the person with dementia (Collaborate questionnaire), experienced level of competence of the FC (sense of competence questionnaire), and health care costs (recourse utilization in dementia questionnaire)	During the 6-month follow-up, ACP was initiated in 35 (49.3%) of the 71 PWD in the intervention group and in 9 (13.9%) of the 65 PWD in the control group (ICC 0.4, OR 1.99; *p* = 0.002) In the intervention group, a total of 165 ACP preferences (58 medical (resuscitation and hospitalisation were common discussions) and 107 nonmedical (housing and care were common discussions) compared to 15 (8 medical and 7 nonmedical) in the control group were documented GPs in the intervention group documented significantly more ACP preferences per patient [mean 2.3, standard deviation (SD) 2.99] than in the control group (mean 0.2, SD 0.7) PWD’s, QoL, PWD’s experienced level of SDM, and the FCs’ sense of competence did not differ between study groups. The cost analysis shows that PWD’s and FCs’ health care costs and PWD’s QALYs did not differ between study groups
Berggren et al. (2016) Sweden [40]	To evaluate the effectiveness of training in nutritional intervention for primary health care professionals practicing in home-based care	87 intervention arm 53 control arm	Cohort study Quasi-experimental	Three phases of intervention: Phase 1 (week 1): web-based program: (1.5 h) Phase 2 (week 2 and 3): practical exercise using mini nutritional assessment tools (home visits): 1 h Phase 3 (week 4) (1.5 h): case-based discussion: reflection on their own case and other’s cases Control group: pre- and post-questionnaire was filled in a gap of 1 month	Survey (pre-post) using Likert scale	As compared to the control group, the interventional group was able to improve self-reported knowledge and confidence in differentiating early from late palliative phase to identify the nutritional needs of the patients Able to distinguish the nutritional needs at early and late palliative phase Advice food and meals that adapt to patient’s requirements and wishes Determining when nutritional needs is no longer needed Confidence in collaboration with other professional caregivers
Hermann et al. (2012) Germany [41]	To evaluate if palliative patients of GPs trained in palliative care have a better health-related QoL	45 GPs IG 27 CG 18 Patients IG 62 CG 34	Quasi-experimental study	Covered topics of symptoms end-of-life care and self-care over 40 h	Quality of life measurement: EORTC QLQ PALL-15 POS ECOG	On the QLQ-C15-PAL, mean QoL of the patient groups of PAMINO-trained and other GPs were 37.7 (SD = 25.5, *n* = 54) and 39.4 (SD = 26.3, *n* = 33) (*p* = .76), respectively. On the POS, respective mean values of 13.6 (SD = 5.8, *n* = 51) and 12.0 (SD = 6.5, *n* = 32) (*p* = .26) were given No difference in physical and emotional functions between groups (on EORTC Pall 15) or palliative outcome scale

**Table 3:** Details of training programs

Domains assessed	References
Eleven studies explored the effect of educational intervention on knowledge	[21, 22, 25–29, 31, 35–37]
Eleven studies explored the effect of educational intervention on skills	[21–28, 35, 36, 39, 40]
Four studies explored the effect of educational intervention on attitude	[22, 24, 29, 31]
Fourteen studies explored the effect of educational intervention on self-efficacy	[21, 22, 25–31, 34–36, 38, 40]
Five studies explored the effect of educational intervention on GPs’ satisfaction	[24, 25, 29, 31, 32]
Twelve studies explored the effect of educational intervention on patient outcomes	[22, 26–28, 30, 32–35, 37, 39, 41]
Nine studies included follow-up post-intervention in determining if the changes were sustained in clinical practice	[21, 23, 24, 26, 27, 33, 35, 38, 39]

Six themes were generated; they were: (1) knowledge translation, (2) skill development, (3) change in attitude, (4) self-efficacy, (5) satisfaction, and (6) patient outcomes (Table 4).

**Table 4:** Impact of the training programs on outcomes

Domain areas	Changes in knowledge	Changes in skills	Changes in attitude	Changes in confidence	GP satisfaction	Patient outcome	Author et al
Identifying palliative needs				+ *			Marshall et al. (2008) [27]
	+ *	+ *					Kadlec et al. (2015) [25]
Identifying and providing				+ *			Thoonsen et al. (2016) [30]
Nutritional interventions		+		+			Berggren et al. (2016) [40]
Collaborating with interprofessional members				+ *			Berggren et al. (2016) [40]
		+					Marshall et al. (2008) [27]
		+		+ *			Kadlec et al. (2015) [25]
			+ *				Shipman et al. (2003) [29]
Pain management		+ *			+		Kadlec et al. (2015) [25]
	+ *			+ *			Landers et al. (2022) [26]
	−	+ *		−		+	Pelayo et al. (2011) [36]
	+						Pelayo − Alvarez et al. (2013) [37]
	+	+		+ *		+	Reymond et al. ( 2005) [28]
			+		+		Thoonsen et al. (2016) [30]
				+			Ward and Walsh (2009) [32]
						+	Abernethy et al. (2013) [34]
						+ *	Xhixha et al. (2013) [33]
Symptom management Advance care planning Communication	+ *	+ *		+ *			Boakes et al. (2000) [21]
				+ *			Evans et al. (2021) [23]
					+	+ *	Marshall et al. (2008) [27]
	+ *			+			Kadlec et al. (2015) [25]
		+					Landers et al. (2022) [26]
		+ *		+ *			Pelayo et al. (2011) [36]
		+ *	+	−			Reymond et al. (2005) [28]
	−				+		Thoonsen et al. (2016) [30]
				+ *			Ward and Walsh (2009) [32]
	+	+		+			Abernethy et al. (2013) [34]
			+ *	+	+ *	+	Xhixha et al. (2013) [33]
				+			Hinkka et al. (2002) [24]
						+ *	Shipman et al. (2003) [29]
	+ *		+	+ *		+ *	Detering et al. (2014) [22]
		+					Evans et al. (2021) [23]
				+ *			Kadlec et al. (2015) [25]
	+ *			+ *			Landers et al. (2022) [26]
		+ *		+ *		+ *	Tilburgs et al. (2020) [39]
	+ *			+ *			Pelayo et al. (2011) [36]
				+ *			Reymond et al. (2005) [28]
				+			Slort et al. (2014) [38]
				+			Ward and Walsh (2009) [32]
Use of surprise question		+					Evans et al. (2021) [23]
Patient’s desire to die at home						+	Marshall et al. (2008) [27]
				+			Slort et al. (2014) [38]
						+	Thoonsen et al. (2015) [31]
						+	Abernethy et al. (2013) [34]
Knowledge on use of local palliative care resources	+ *						Kadlec et al. (2015) [25]
	+ *						Shipman et al. (2003) [29]
Ethical aspects				^−^			Landers et al. (2022) [26]
Patient/caregiver satisfaction						+	Pelayo-Alvarez et al. (2013) [37]
Quality of life of patients						^−^	Tilburgs et al. (2020) [39]
						+	Hermann et al. (2012) [41]
Coping with managing dying patients				+			Ward and Walsh (2009) [32]
			+		+ *		Hinkka et al. (2002) [24]

^+^Change observed

^−^No change seen

^*^Statistically significant

The findings of this review showed that GPs accessed diverse end-of-life care training programs. They were web-based learning [24, 30, 31, 34, 36, 37, 39, 40], didactic seminars [21, 24, 28, 33, 41], small-group interactive workshops [22–28, 30–32, 38, 39], simulated learning environment [22, 38, 39], reflective learning through trigger cases [21, 24, 26, 27, 32, 40], mentor-facilitated experiential learning [21, 24, 25, 32, 40], self-directed learning [22, 35–37, 39, 40], learning through peer collaboration [23, 27, 40], participation in reviews [24, 27], and role-play [24]. In most studies (17 out of 21), GPs were exposed to more than one kind of end-of-life care training programme, and the learning period whilst accessing them ranged from a minimum of 3 h to a maximum of 2 years. Longer training interventions covered a broad range of topics like identification of patients with palliative care needs, pain and symptom management, nutritional support, interprofessional collaborative care, advance care planning, communication, bereavement care, ethical aspects of care, knowledge of local palliative care resources, self-care, and coping with death and dying.

### Knowledge Translation

Knowledge translation is an ongoing dynamic process that includes knowledge construction through social interaction, dissemination of information, and ethical application of this knowledge to improve the health and well-being of patients [42]. It also aids in strengthening the healthcare system through the effective delivery of healthcare services [42]. In the knowledge translation process, it is essential to consider the interrelationship between context, relevance, meaning, evidence, and cost [42].

Evidence from studies included in this review suggests that knowledge improvement was mainly determined using the pre-post-test questionnaire [22, 25–29, 31, 36], followed by the GP’s self-perception of change in knowledge post-intervention [21, 25, 37]. Eleven studies explored the effect of end-of-life care training programs on knowledge enhancement. Training that included a combination of small-group interactive workshops, mentor-facilitated experiential learning, and reflective learning through trigger cases demonstrated significant improvement in the GPs’ knowledge in the following domains: identification of patients with palliative care needs[27], pain [26, 28, 37] and symptom management [21, 25, 31], communication [36], advance care planning [22, 25], bereavement care [35], and knowledge on local palliative care resources [25, 29]. Combining multiple learning methods and a longer training duration showed better education outcomes regarding gain in end-of-life care knowledge.

### Skill Development

Skill is an individual’s potential to coordinate the acquired knowledge into performance efficiently [43]. The skilful transformation of knowledge into practice requires internal personal assets, motivation, goal setting, emotional control, self-esteem, and work ethics [43].

Evidence from studies included in this review suggests that the GP’s self-perception about change in skills post-intervention was the most common assessment [21, 23, 25, 37, 39], followed by a pre-post-test questionnaire [22, 26–28, 35]. Fourteen studies explored the effect of educational intervention on skills acquired post-intervention. Training programs that included a combination of small-group interactive workshops, mentor-facilitated experiential learning, reflective learning through trigger cases, and learning through peer collaboration demonstrated significant improvement in the GPs’ skills in identifying patients with palliative care needs [27], pain [25, 26] and symptom management [21, 26, 36], advance care planning [39], bereavement care [35], interprofessional collaboration [25, 27], and the use of a surprise question [23]. Combining multiple learning methods and a longer training duration showed better education outcomes regarding the GPs’ skill enhancement.

### Change in Attitude

Attitude is a cultivated habit or a mental state of preparedness to act consistently towards a context or a situation [44]. Attitudinal shifts and behavioural change can be complex and mediated by learners’ age, years of experience, personal disposition, and organisational factors [44].

The studies assessed attitude using a pre-post-test questionnaire [24, 29, 31] and the GP’s self-perception [22, 30]. Training programs that ranged from 3 h to 1 year and that used a combination of small-group interactive workshops, reflective learning through trigger cases, a simulated learning environment, and self-learning demonstrated a significant improvement in the participants’ attitudes towards interprofessional collaborative work [29], symptom management [24], pain [31], advance care planning [22], and coping with managing dying patients [24].

### Self-Efficacy

Self-efficacy is the individual’s ability to perform a task to accomplish desired goals [45]. The belief in one’s capability and the dynamic interplay between environmental and behavioural factors to achieve the task influence the choice of action, level of effort, and persistence, which have the potential to inform practices [45].

Findings from the studies included in this review suggest that the GPs’ self-efficacy was assessed using a pre-post-test questionnaire [23, 25, 26, 28, 29, 32, 34, 35, 38, 40] followed by the GPs’ self-perceived confidence [22, 27, 31, 37] and pre-post-test questionnaire [28, 40]. Fifteen studies investigated the effect of training programs on GPs’ self-efficacy in delivering end-of-life care. Training programs that used a combination of small-group interactive workshops, simulated learning environments, learning through peer collaboration, and self-learning demonstrated significant improvement in self-efficacy in the following domains: nutritional intervention [31], pain [32, 36] and symptom management [25, 28, 29, 32, 34], communication [32, 38], fulfilling patients’ desires to die at home [38], coping with managing dying patients [32], and ethics in end-of-life care [26]. The duration of the intervention ranged from a minimum of 3 h to a maximum of 24 months. Combining multiple learning methods and a longer training duration showed better education outcomes regarding the GPs’ self-efficacy.

### Satisfaction

Satisfaction is an individual’s subjective appraisal of the training program to evaluate if the training experience aligns with academic expectations [46]. Many variables are associated with the subject’s satisfaction, such as the value attributed to the context of the training programme, the relevance of the training to clinical practice, teaching strategies, the learning environment, and faculty expertise [46].

Data from the studies included in this review suggests that the GPs’ self-perceived satisfaction was used to determine satisfaction with training [24, 25, 29, 31, 32]. Five studies investigated the GPs’ satisfaction with the educational intervention. There was wide variation in the duration of training programs, ranging from a minimum of 5 h to a maximum of 24 months. The GPs were satisfied with the training programs when they combined small-group interactive workshops, reflective learning through trigger cases, mentor-facilitated experiential learning, and regular follow-up and feedback [24, 25, 29, 31, 32].

### Patient Outcomes

Patient-reported outcomes include multidimensional and subjective feedback grounded in the patients’ perceptions, which are then objectively quantified [14]. These capture patient feedback on symptom control, their feelings, the experience of the clinical journey, and the effects of prescribed treatment as a measure of quality care delivery [14]. It will ensure a safer practice environment for patients and providers and improve patient safety [14].

Evidence from studies included in this review suggests patient outcomes were measured using a questionnaire-based survey [30, 36, 41] and self-reported scales [27, 35]. Training programs that used a combination of small-group interactive workshops, reflective learning through trigger cases, and learning through peer collaboration reduced hospital utilisation at the end-of-life [34]. The interventions also enabled GPs to fulfil the patient’s desire to die at home at the end-of-life [27, 30, 34]. Furthermore, combining interventions that included online training, self-learning, a small-group interactive workshop, and a simulated learning environment improved patient outcomes regarding the control of pain and psychological symptoms and enhanced the quality of life [34–36, 41]. These interventions also improved the discussion and documentation of advance care planning [39]. Also, improving the GP’s knowledge and skills in end-of-life care enhanced patients’ and caregivers’ satisfaction [41]. There was wide variation in the duration of the intervention, ranging from a minimum of 5 h to a maximum of 15 months; however, combining multiple learning methods and a longer training duration showed better education outcomes in terms of patient outcomes.

## Discussion

Contemporary training programs are rapidly adopting blended learning modules [47]. Blended learning uses a mix of instructional designs and delivery modes and combines traditional face-to-face, distance, and self-paced learning [47]. It is the preferred mode of delivery due to the ease of delivery concerning time and pace [47]. Face-to-face interaction helps reduce feelings of isolation and the likelihood of learners losing interest [47]. The blended learning in the reviewed studies included small-group interactive workshops, reflective learning through trigger cases, mentor-facilitated experiential learning, mentored online training, and learning through peer collaboration. The blended learning in the reviewed studies demonstrated significant improvements in the GPs’ knowledge, skills, attitude, and self-efficacy in delivering end-of-life care. These interventions also enabled GPs to alleviate the patients’ physical and psychological symptoms, reduce hospital utilisation rates, fulfil the patients’ desire to die at home at the end-of-life, mitigate caregiver anxiety, and enhance their satisfaction.

A recent systematic review explored the GP’s preference for end-of-life care learning [10]. It revealed that GPs preferred a multi-modal approach to learning, with self-learning, reflective, and experiential learning as the preferred styles [10]. Their learning preferences varied with personal disposition, professional and organisational challenges, and their relationship with the specialist palliative care teams or GP colleagues experienced in end-of-life care [10].

The findings of our review corroborated the results of a study by Lennaerts-Kats et al., which showed that a blended-learning programme positively impacted knowledge acquisition and improved collaboration between primary care physicians and palliative care teams [48]. Furthermore, elderly GPs, GPs with many years of clinical practice experience, GPs with a higher clientele of end-of-life care patients, and those involved in group practice were highly motivated to undergo training in end-of-life care and had a higher likelihood of attending the training programs [10, 48]. It could explain the significant improvement in knowledge and interprofessional collaboration with the palliative care teams after the training [48]. Three other studies mirrored the findings in our review [49–51]. The studies investigated the effect of digital and experiential learning and reflective learning on healthcare professionals’ attitudes, self-efficacy, and skills [49, 50]. The interventions changed the healthcare professionals’ attitudes towards end-of-life patients [49], mitigated their fear [49–51], improved their self-efficacy in managing difficult communication [49–51], and enhanced their skills in managing physical and psychological symptoms [51] and handling difficult communication [49, 50].

Blended learning allows learners to flexibly utilise the tools according to their needs [47]. The e-learning component enables learners to explore sensitive issues surrounding death and dying, reduces stigma or judgement, and increases accessibility to a larger group of healthcare professionals, especially for those accessing from rural and remote areas [52, 53]. The face-to-face interaction component allows learners to discuss issues with colleagues and course facilitators, adds depth to their understanding through a mutual exchange of views, feelings, and ideas and facilitates the legitimate integration of knowledge into practice [52, 53]. Despite training, GPs were apprehensive about using syringe drivers, discussions surrounding advance care planning, or resolving ethical dilemmas [26]. Mentor-facilitated experiential learning, reflection on trigger cases, and learning through peer collaboration improved their self-efficacy and skills in managing the challenges mentioned above [54, 55]. Also, learning through peer collaboration on an ongoing basis resulted in the early initiation and proactive documentation of advance care planning [22, 23, 26, 39]. Learning through peer collaboration is also known to benefit GPs who have a solo practice, work in a resource-constrained setting, or work in remote or rural areas [23, 26]. Learning through peer collaboration can strengthen palliative care delivery as this exposes the learner to diverse clinical approach to caring for patients, thereby increasing their understanding of the critical role that each member in the team plays in providing palliative care [56, 57]. Training programs must be ongoing to drive behavioural change [23, 26, 48]. Also, the learners must have the opportunity to receive regular follow-ups and feedback from mentors and GP champions [23, 26, 48]. It will ensure knowledge and skill retention and continuous performance improvement [23, 26, 48]. The follow-up that includes reflection on one’s practice will enhance the knowledge, skill, and self-efficacy of the GPs and translates into better quality care [23, 26, 48].

A systematic review of training programs in end-of-life care showed that training had beneficial effects on physician-reported self-efficacy and attitude towards palliative care but had limited impact on their performance and patient outcomes [30, 58]. Patient-reported outcomes offer an evidence-based approach to detecting symptoms, which can provide critical information to healthcare professionals and improve healthcare delivery [59]. The patient-reported outcome is the reporting of a patient’s status that comes directly from the patient, without the interpretation of the patient’s response by a healthcare professional [59]. Integrating patient-reported outcomes in clinical practice acts as an aid to clinicians in monitoring patient symptoms, identifying unmet needs and concerns, and prioritising and tailoring the treatment to individual needs [60]. Additionally, it can foster communication between healthcare professionals and patients, assist in discussions surrounding disease progression and end-of-life care, and facilitate optimal delivery of end-of-life care [60]. Patient-reported outcomes trigger proactive identification of symptoms, improve the patient’s physical function, reduce caregiver dependence, promote better symptom control, reduce emergency room visits and subsequent hospitalisations, improve patient satisfaction, and are cost-effective [34, 59, 60].

### Limitations and Strengths 

A few studies included in this review had a mixed population of general practitioners and nurses working in a community setting [28, 36, 37]. It was challenging to disaggregate their views. Studies were restricted to training programs for end-of-life care in general practice. General practitioners trained in a hospital setting were excluded. Furthermore, the synthesis was limited to published end-of-life care training programs. Although studies did mention the use of a combination of modes of training delivery, there was no rationale for using a particular approach. Also, the duration of the interventions varied, ranging from a minimum of 3 h to a maximum of 24 months. Concluding the effect of a particular combination of the mode of delivery and the duration of the training programs on outcome measures was difficult.

The strength of the scoping review is the use of a comprehensive search strategy based on a broad research question. The reviewers used a systematic method to conduct the study using a robust criteria-based selection of literature. Furthermore, the methodological rigour of the review was enhanced using the PAGER framework.

### Implications for Policy and Research

Learners’ preference for a particular learning style depends on their learning needs and varies with the learning environment [61]. It may be essential for educators to conduct a pre-test evaluation that will explore the learner’s knowledge, their learning needs, and their preferences for learning styles. It will help educators determine the learning objectives and design training programs based on the learners’ preferences. For training programs to bring discernible improvement in the GPs’ performance, the programs must be conducted over an extended period. Furthermore, the programs must include regular contact sessions with palliative care specialists and have an in-built feedback and reflective learning mechanisms. Patient-reported outcomes are vital and are known to improve healthcare delivery [14, 60]. Future training programs and research must incorporate patient feedback about end-of-life care delivery by GPs as one of the outcome indicators, as there is very little information on the patients’ confidence and satisfaction with GP intervention.

## Conclusion

The review demonstrated that a blended-learning approach that combined small-group interactive workshops, reflective learning through trigger cases, mentor-facilitated experiential learning, mentored online training and learning through peer collaboration with regular follow-up post-training significantly improved the GPs’ knowledge, skills, attitude, and self-efficacy in providing end-of-life care. The training programs also translated into better patient outcomes and patient and caregiver satisfaction. This review also contributed to new knowledge by exploring the effect of the training programme on patient-reported outcomes, as patient-reported outcomes contribute to quality healthcare delivery and enhance patient safety [60].

### Supplementary Information

Below is the link to the electronic supplementary material.

Supplementary file1 (PDF 126 KB)

Supplementary file2 (PDF 40 KB)

## Data Availability

Summary of the results is attached.
