# Supplementary material for: End-of-Life Care Education as Blended Learning Approach for General Practitioners: a Scoping Review
Source: J Cancer Educ. 2023 Aug 30;38(5):1440–58. doi: 10.1007/s13187-023-02358-w (PMC10509089; doi:10.1007/s13187-023-02358-w)
Supplement: Supplementary file 1 — Supplementary file1 (PDF 126 KB) [file 13187_2023_2358_MOESM1_ESM.pdf]

## Supplementary File 1: Search Terms

| | PUBMED | CINAHL | PsycINFO | EMBASE | Free text |
|---|---|---|---|---|---|
| | MeSH terms | MH terms | DE terms | Emtree terms | |
| **Concept 1**<br><br>**General Practitioner** | 1.General practice<br>2.General practice physician<br>3.Family physician<br>4.General practitioner<br>5.Family practice | 1.General practitioner<br>2.Family physician<br>3.Primary care physician | 1.Family Medicine<br>2.Family Physician | 1.General practitioner<br>2.Primary care physician<br>3.Family physician<br>4.General practice physician<br>5.Primary care physician<br>6.General practice | 1."General practice*"<br>2."General practitioner*"<br>3."Family physician*"<br>4."Family practice* |
| **Concept 2**<br><br>**Continuing medical education** | 1.Continuing education<br>2.Continuing medical education | 1.Continuing medical education<br>2.Continuing education<br>3.education | 1.Continuing education<br>2.Medical education<br>3.Professional development<br>4.Distance education | 1.Continuing medical education<br>2.Continuing education<br>3. Continuing professional development | 1. Continuing medical education<br>2. Continuing education<br>3. Lifelong learning<br>4. life-long learning |
| **Concept 3**<br><br>**End-of-life care** | 1.Palliative care<br>2.Palliative medicine<br>3.Hospice<br>4.Hospice care<br>5.Hospice program<br>6.Terminal care<br>7.End of life care | 1.End of life care<br>2.Terminally ill patient 3.palliative care | Palliative care | 1.Terminal care<br>2.Palliative therapy<br>3.Hospice care<br>4.End of life care<br>5.Terminal care<br>6.Palliative care<br>7.Palliative Medicine<br>8.Hospice care<br>9.Hospice | 1.Palliat*<br>2.Hospice*<br>3.”End of life care"<br>4."supportive care" |
| **Concept 4**<br><br>**Training outcome** | 1.Assessment, patient outcome<br>2. Assessment, patient outcomes<br>3. Assessment, outcome healthcare<br>4. Assessment, outcomes healthcare | 1. Patient outcomes<br>2. Quality of care<br>3. Health outcomes<br>4.Patient satisfaction<br>4.Patient experience<br>5. Patient outcome assessment | 1.Treatment outcome<br>2.Healthcare outcome assessment | 1.Treatment outcome<br>2.Healthcare outcome assessment | 1.Patient outcome*<br>2. Patient feedback<br>3. Feedback<br>4. Assessment*<br>5. Quality of care<br>6.Patient satisfaction<br>7.Patient experience* |
