# Supplementary material for: End-of-Life Care Education as Blended Learning Approach for General Practitioners: a Scoping Review
Source: J Cancer Educ. 2023 Aug 30;38(5):1440–58. doi: 10.1007/s13187-023-02358-w (PMC10509089; doi:10.1007/s13187-023-02358-w)
Supplement: Supplementary file 2 — Supplementary file2 (PDF 40 KB) [file 13187_2023_2358_MOESM2_ESM.pdf]

Supplementary File 2: Types of interventions for General Practitioners in End-of-Life care

**Author/Study**	**Online training Under mentorship**	**Workshops/ Seminars Didactic**	**Workshop/ Seminar Interactive group discussion**	**Simulation**	**Reflecting on Case/problem**	**Experiential learning under mentorship**	**Self-learning**	**Learning from collaborative work**	**Reviews/ audits**	**Role play**	**Duration of intervention**
Berggren et al. (2016)	+				+	+	+	+			4weeks
Boakes et al. (2000) Australia		+			+	+					6months
Detering et al., (2014) Australia			+	+			+				3hours
Evans et al., (2021) Canada			+					+			2days
Marshall et al., (2008) Canada			+		+			+	+		15months
Guldin et al., (2013) Denmark							+				13months
Kadlec et al., (2015) Canada			+			+					4months
Landers et al., 2022			+		+						2years
Pelayo et al., 2011	+						+				75days

Supplementary File 2: Types of interventions for General Practitioners in End-of-Life care

Pelayo-Alvarez et al., 2013	+						+				75days
Reymond et al., 2005		+	+								3hours
Slort et al., 2014			+	+							6months
Thoonsen et al., 2016	+		+								5hours
Thoonsen et al., 2015	+		+								5hours
Tilburgs et al., 2020	+		+	+			+				6months Monthly F/U
Ward and Walsh 2009			+		+	+					-
Abernethy et al., 2013	+					+					4weeks
Hermann et al., 2012		+									40hours
Xhixha et al., 2013		+									8hours
Hinkka et al., 2002	+	+	+		+				+	+	1year
Shipman et al., 2003						+					2years
